# Sexual Conflict and Sexually Antagonistic Coevolution in an Annual Plant

**DOI:** 10.1371/journal.pone.0005477

**Published:** 2009-05-07

**Authors:** Josefin A. Madjidian, Åsa Lankinen

**Affiliations:** Department of Plant Ecology and Systematics, Lund University, Lund, Sweden; McGill University, Canada

## Abstract

**Background:**

Sexual conflict theory predicts sexually antagonistic coevolution of reproductive traits driven by conflicting evolutionary interests of two reproducing individuals. Most studies of the evolutionary consequences of sexual conflicts have, however, to date collectively investigated only a few species. In this study we used the annual herb *Collinsia heterophylla* to experimentally test the existence and evolutionary consequences of a potential sexual conflict over onset of stigma receptivity.

**Methodology/Principal Findings:**

We conducted crosses within and between four greenhouse-grown populations originating from two regions. Our experimental setup allowed us to investigate male-female interactions at three levels of geographic distances between interacting individuals. Both recipient and pollen donor identity affected onset of stigma receptivity within populations, confirming previous results that some pollen donors can induce stigma receptivity. We also found that donors were generally better at inducing stigma receptivity following pollen deposition on stigmas of recipients from another population than their own, especially within a region. On the other hand, we found that donors did worse at inducing stigma receptivity in crosses between regions. Interestingly, recipient costs in terms of lowered seed number after early fertilisation followed the same pattern: the cost was apparent only if the pollen donor belonged to the same region as the recipient.

**Conclusion/Significance:**

Our results indicate that recipients are released from the cost of interacting with local pollen donors when crossed with donors from a more distant location, a pattern consistent with a history of sexually antagonistic coevolution within populations. Accordingly, sexual conflicts may have important evolutionary consequences also in plants.

## Introduction

Sexual conflict is tacitly believed to concern the differing interests of a male and a female during reproductive interactions, whereas it is in fact a conflict between two reproducing individuals, whether these are unisexual or hermaphroditic [1]–[4]. Sexual conflict theory predicts that male and female sexually antagonistic traits will coevolve as each reproducing individual tries to increase its own fitness at the expense of another [5]–[8, see also review 1]. Sexually antagonistic coevolution has for example been proposed in *Drosophila melanogaster*, where experimentally imposed monogamy resulted in evolution of males that were less harmful to females, and females that were less resistant to male-induced harm [9]. Even though sexually antagonistic coevolution has the potential to be as important as local adaptations and genetic drift for population differentiation and speciation [7], [10]–[12], most empirical studies conducted to date have collectively investigated only a few species (i.e. insects, reviewed in [2], but e.g. see [3], [13] for sexually antagonistic coevolution in hermaphroditic animals).

In recent years, an increasing number of researchers have suggested that sexual conflicts could occur in plants [14]–[21], but empirical evidence is still scarce. One possible conflict scenario in plants is a conflict over parental investment during seed provisioning, as paternal genes should favour higher levels of investment in the seeds than maternal genes [22]–[26]. Further, conflicts over mating and fertilisation in plants have been suggested to be important during processes in the prezygotic stage [16], [27], [28], that is, after pollination but before fertilisation. For example, Lankinen et al. [17] showed in a model that floral wilting, which could be due to several different factors (see e.g. [29]), may also be the cause of a prezygotic conflict, where pollen induces the flower or stigma to wilt in order to minimize the risk of competition by later arriving pollen (cf. “defence ability” in sperm; [30]–[31]). In a recent empirical study, we investigated the occurrence of a sexual conflict over timing of stigma receptivity in *Collinsia heterophylla* [20]. We found that both recipient and donor affected timing of stigma receptivity, implying that some donors can fertilise an ovule ahead of others (cf. “offense ability” in sperm; [30]–[31]). Early fertilisation resulted in fewer seeds, suggesting a cost experienced by the female sexual function [20]. Even though donors that produce early-germinating pollen will also be affected by this cost, the ability to induce stigma receptivity could still be selected for if this trait results in a higher fertilisation success (e.g. by securing paternity) [cf. 10]. Because these results are consistent with a sexual conflict, further studies have the potential to generate important knowledge of the generality of sexual conflict theory. In the present study we aimed at exploring the potential evolutionary consequences of this possible sexual conflict in populations of *C. heterophylla* originating from two distant geographic regions.

It is probable that intersexual coevolution will take different pathways in geographically separated populations of the same species due to stochastic processes and differences in selection history [11], [32]. Therefore, crossing experiments involving both intra- and inter-population matings is a possible approach to test whether sexually antagonistic coevolution acts within populations [e.g. 8], [11], [32]–[35]. As individuals of a population may not have evolved counter adaptations to sexually antagonistic traits of other populations, they are expected to be more responsive (adapted) to sexually antagonistic traits of their own and closely related populations, while being less responsive to more distantly related populations [8], [11], [32]. As a result, it should be more costly being fertilised by a foreign individual. Inter-population crosses in insects following such reasoning have, however, yielded inconsistent results [reviewed in 36], which has led to criticism of this experimental approach [e.g. 37]–[39]. Rowe et al. [38] showed theoretically that statistical interactions between populations are not diagnostic of a sexually antagonistic coevolution and that females should not always perform better with coevolved males. One explanation could be that populations may be at different sexually antagonistic coevolutionary stages, i.e. either the male or the female are in the advantageous position [2], obstructing the outcome of inter-population crosses. Furthermore, interactions between populations could be confounded by the degree of divergence between populations used in the crossing experiment. Indeed, one problem with the inter-population cross approach has been the lack of information on population history and genetic divergence between populations [11], [37], [41], [but see 40], [42]. Compared to recently isolated populations, incipient species may be so differentiated in their reproductive or sexual characters that a sexually antagonistic trait will not be effective in another population. It would therefore be of interest to explore inter-population crosses involving populations of different relatedness. Because it is possible that more distantly related populations could have either less or more effective sexually antagonistic traits, it is further crucial to study if the mating success of the sexually antagonistic trait covaries with the cost inflicted on the mating partner. Absence of covariance between the mating success and the cost inflicted on the mating partner would thus suggest an absence of sexually antagonistic coevolution. In the present study on *C. heterophylla* we performed intra- and inter-population crosses using four populations from two different regions. By conducting crosses within and between both populations and regions, we were able to investigate recipient-pollen donor interactions at three levels of geographic distances between interacting individuals. It is conceivable that the more distant populations show a higher degree of differentiation [43], especially as the two regions are located in an area well known for its high rate of diversification and speciation along mountain ranges [44]–[45].

Other confounding effects that might appear in between-population crosses are outbreeding depression, inbreeding-avoidance or heterosis (higher quality offspring when fertilised by pollen donors from other populations) [46]–[48]. Because we were able to focus on both a possible male sexually antagonistic trait (pollen germination on a not completely receptive stigma) and a possible female fitness cost (reduced seed set after early fertilisation) [20] it should be easier to exclude such effects (e.g. we would generally not expect lowered seed set only at early fertilisation).

In this study on *Collinsia heterophylla* we conducted one-donor crosses between and within populations and regions in order to investigate (1) the generality of our previous result from one population [20], i.e. whether certain pollen donors are consistently better at inducing stigma receptivity than other donors. We further asked (2) whether the geographic distance between populations serving as pollen donor and recipient affects the onset of stigma receptivity, and (3) whether population proximity influence recipient cost in terms of lowered seed set at an early fertilisation. In order to get an indication of the degree of population differentiation we further investigated (4) whether the experimental populations differ in timing of stigma receptivity and other characters. Timing of stigma receptivity has been shown to be positively associated with timing of self-pollination, a floral trait related to the mating system, across the genus *Collinsia* [49]. As such correlations may indicate a genetic covariance, constraining independent selection on timing of stigma receptivity [50]–[51], we additionally asked (5) whether timing of self pollination is correlated with timing of stigma receptivity.

## Results

### Onset of stigma receptivity following crosses within or between populations of two regions

In the analysis taking recipient/donor and recipient/donor population of origin into account (Analysis 1), both recipient and pollen donor identity affected start of stigma receptivity. There was no significant recipient×donor interaction. This result thus indicates that some pollen donors were consistently better than others at fertilising a partly receptive stigma across all four populations. At the population level we observed a non-significant trend for an effect of the interaction between recipient and pollen donor (Table 1).

**Table 1:** Effects of recipient and donor population on onset of stigma receptivity.

Source of variation	df	*F*	*P*
Recipient population	3	2.25	0.13
Pollen donor population	3	0.828	0.50
Recipient population×Pollen donor population	9	1.91	0.071
Recipient (population)	22	2.70	0.001
Pollen donor (population)	12	2.52	0.01
Recipient×Pollen donor (population)	54	0.739	0.89
Error	107		

Nested, factorial random-effect ANOVA for onset of stigma receptivity following one-donor crosses performed within or between four populations of *Collinsia heterophylla* (Analysis 1).

**Figure 1:**
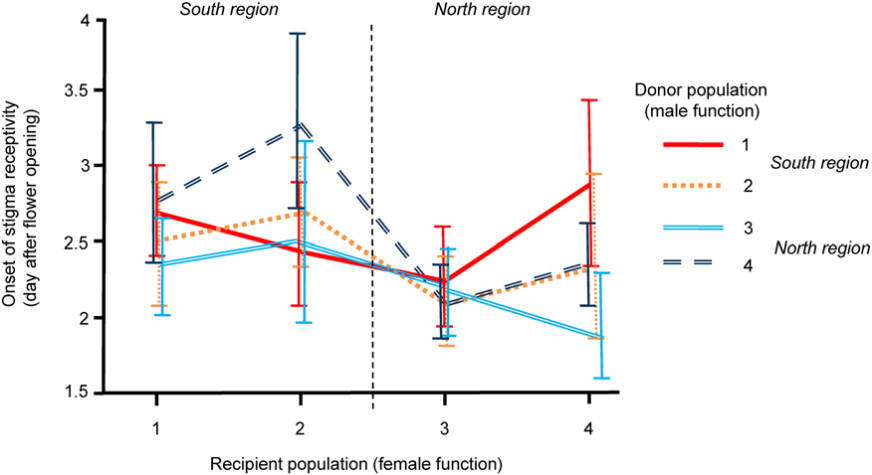
Mean onset of stigma receptivity following crosses within/between populations and regions. Error bars indicate standard error.

**Figure 2:**
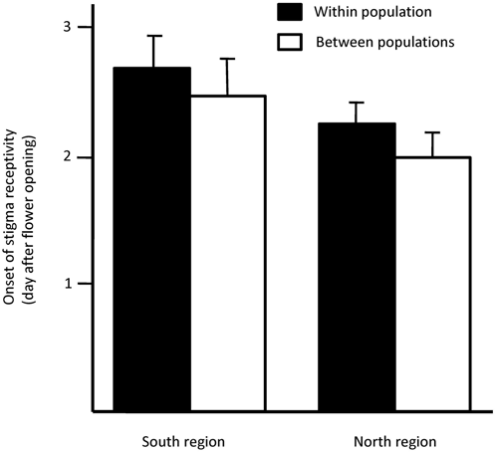
Mean onset of stigma receptivity following crosses within/between populations for each region. Note that these results represent a subset of the results shown in Figure 1. Error bars indicate standard error.

When analysing the effects of cross type (within/between populations) and the region from which the recipient and donor originated (Analysis 2), we found a significant effect of cross type (Table 2). Crosses between populations in general resulted in *earlier* start of stigma receptivity, indicating that pollen donors had a greater success at inducing stigma receptivity if the recipient did not belong to the same population as the donor. However, the difference was very small (mean±S.E. of developmental stage, between populations 2.42±0.068, within populations 2.46±0.076). Another significant, and potentially more important, effect influencing timing of stigma receptivity was the interaction between the regional origin of the recipient and donor (Table 2). In crosses between regions, timing of stigma receptivity appeared *later* compared to crosses within regions, i.e. donors were less capable of inducing stigma receptivity on recipients originating from another region (Figure 1). A separate test involving only within-region crosses showed that within each region onset of stigma receptivity was instead *earlier* in crosses with foreign but closely related pollen rather than with pollen from the same population (two-way ANOVA: region (random) *F*_1,1_ = 1100, *P* = 0.019, cross type (own/other population within region) *F*_1,1_ = 311, *P* = 0.036, region×cross type *F*_1,135_ = 0.010, *P* = 0.92; Figure 2).

**Table 2:** Effects of recipient region, donor region and cross type (within/between populations) on onset of stigma receptivity.

Source of variation	df	*F*	*P*
Recipient region	1	2.65	0.51
Pollen donor region	1	0.030	0.89
Within/between populations	1	310	0.036
Recipient region×Pollen donor region	1	4.18	0.042
Recipient region×Within/between populations	1	0.10	0.92
Error	205		

Factorial ANOVA for onset of stigma receptivity following one-donor crosses performed within or between four populations of *Collinsia heterophylla* originating from two regions (Analysis 2).

### Seed production following crosses within or between regions

**Figure 3:**
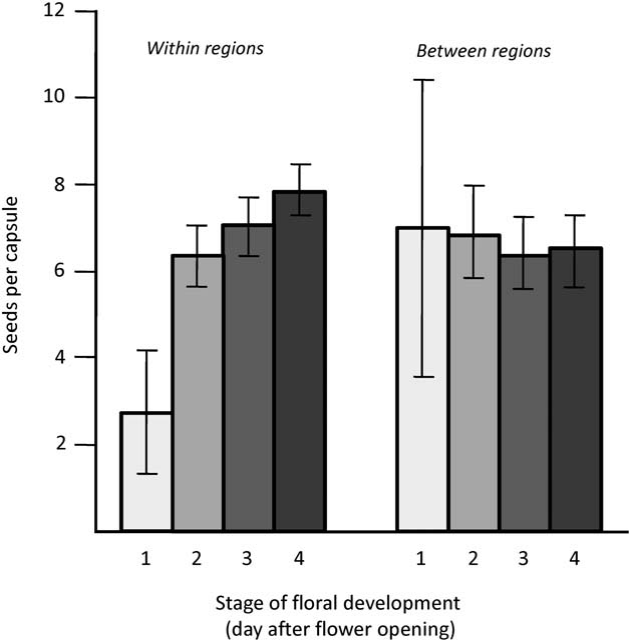
Mean seed set following crosses at different floral developmental stages (1–4). Pollen donors were either of the same population or region as the recipient (within regions), or of populations of the other region (between regions). Error bars indicate standard error.

In the experimental crosses, significantly fewer seeds were produced after pollination at an early developmental stage if the pollen source was from the same region as the recipient compared to if the pollen source was from another region (Table 3, Figure 3). Fertilisation during early floral development may thus be costly for these recipients in terms of lowered seed production. No such recipient costs existed if the pollen donor originated from the other region (Table 3, Figure 3). We found no main effect of cross type on seed production, i.e. if crosses were conducted between or within regions (Table 3).

**Table 3:** Effects of stage of floral development and cross type (within/between regions) on seed set.

Source of variation	df	*F*	*P*
Floral development stage	3	1.97	0.117
Within/between regions	1	1.46	0.23
Floral development stage×Within/between regions	3	3.80	0.010
Error	502		

Two-way ANOVA for number of seeds after one-donor crosses within and between four populations of *Collinsia heterophylla* originating from two regions.

### Variation among greenhouse-grown populations

Pollen tube growth rate *in vitro* was faster, and flowers were larger in Southern region populations than in Northern region populations (Table 4). There were no other significant differences between regions; however there was a general trend that flowers of Southern region populations started to flower later, and had later timing of anther-stigma contact and later onset of stigma receptivity (Table 4). Within regions, populations significantly differed in general measures of sporophytic fitness, but not in pollen tube growth rate, timing of stigma receptivity, and other traits presumably connected to the mating system (flower size and timing of anther-stigma contact). Some variables, especially timing of stigma receptivity, showed large variation within populations (Table 4).

**Table 4:** Traits related to general fitness and mating system.

	Southern region		Northern region		*P*_pop (region)_	*P*_region_
Character	Pop 1	Pop 2	Pop 3	Pop 4		
Prop. seeds germinated^a^	0.95±0.21	0.88±0.30	0.53±0.18	1.0±0.26	<0.001	0.61
	N = 7	N = 13	N = 14	N = 11		
Start of flowering (day^b^)	8.14±1.47	6.63±3.91	4.09±2.59	1.92±2.57	0.041	0.78
	N = 8	N = 13	N = 14	N = 12		
Number of shoots	9.3±1.7	8.4±1.6	8.5±1.7	6.8±1.8	0.019	0.36
	N = 7	N = 14	N = 17	N = 13		
Pollen tube growth rate *in vitro* (mm/1.75h)	17.5±2.10	17.0±3.89	15.9±2.10	15.7±3.48	0.92	0.004
	N = 6	N = 8	N = 21	N = 9		
Flower size (mm)	18.8±0.77	18.9±1.50	17.82±2.55	16.71±1.24	0.26	0.005
	N = 7	N = 14	N = 16	N = 13		
Timing of anther-stigma contact (stage^c^)	3.18	3.37	2.94	2.79	0.11	0.17
	(2.95–3.38)	(3.08–3.54)	(2.81–3.08)	(2.50–2.94)		
	N = 7	N = 14	N = 16	N = 12		
Timing of stigma receptivity (stage^c^)	2.41	2.13	2.06	2.00	0.56	0.23
	(2.06–2.67)	(1.52–2.37)	(1.33–2.35)	(1.41–2.34)		
	N = 8	N = 13	N = 13	N = 12		

Means (and standard deviations) of traits related to general fitness and mating system for four populations of *Collinsia heterophylla* originating from two regions. For onset of stigma receptivity and timing of anther-stigma contact population estimates were calculated as the floral developmental stage (and 95% confidence interval) when 50% of the plants had receptive stigmas or stigmas contacting the anthers, respectively. N = number of maternal families/plant individuals. Differences between populations and regions were tested for significance using ANOVA with population nested within region (means of maternal families/plant individuals represent individual data points in this analysis).

a Arcsine transformed.

b The day the first plant started flowering represents day 1.

c Stage 0 = day of flower opening, stage 1-4 equals the number of dehisced anthers (one per day).

With our limited sample of populations, it was not possible to detect a significant positive association between timing of stigma receptivity and timing of anther-stigma contact across populations, despite a trend in the expected direction (Pearson r = 0.547, df = 2; *P* = 0.45). Among individual plants, however, these traits appeared unrelated (ANCOVA: population *F*_3,38_ = 1.46, *P* = 0.24; timing of anther-stigma contact *F*_1,38_ = 0.91, *P* = 0.35).

## Discussion

In this study on *Collinsia heterophylla* we found that onset of stigma receptivity not only was affected by the identity of the individual serving as recipient or pollen donor, but also by the geographic distance between the populations from which the recipient and donor originated. Pollen donors were generally better at inducing stigma receptivity on recipients belonging to another population than their own. On the other hand, donors were less capable of inducing stigma receptivity in crosses between different regions than in crosses between populations belonging to the same region. Interestingly, recipient costs in terms of lowered seed production at early fertilisation showed a similar pattern in that the cost was present in crosses within regions and populations, but absent in crosses between regions. These results suggest that recipients are released from the cost of interacting with local pollen donors when crossed with more distant pollen donors, indicating the existence of sexually antagonistic coevolution within populations of *C. heterophylla*.

Empirical evidence of sexual conflicts in plants is still sparse and the information that exists is scattered [16], [19], [20]. In a previous study on *C. heterophylla* [20] we identified a possible sexual conflict over timing of stigma receptivity. In the current study we aimed at evaluating the evolutionary consequences, such as sexually antagonistic coevolution potentially leading to population differentiations, of this conflict by performing inter-population crosses. The use of several populations also allowed us to investigate the generality of our previous results, which referred to a single population. Because all of our four populations consistently followed the same pattern previously found, i.e. that some pollen donors were better than others at inducing stigma receptivity, which in turn inflicted a cost on recipients [20], we conclude that the potential sexual conflict we have identified appears to be general. However, it should be noted that we have only performed one-donor crosses, so we can not be sure about how the seemingly advantageous pollen donors would have performed in cases of more intense pollen competition. For example, it is possible that the benefit of inducing stigma receptivity may be reduced if this manipulation also facilitates the germination of other pollen grains [cf. 17].

Recent inter-population cross experiments on insects have used populations differentiated in the lab to control for population history [40], [52]–[53]. For example, using *Drosophila melanogaster*, Long et al. [40] performed inter-population crosses between six replicated laboratory strains originating from one ancestral population that had been maintained in similar culture conditions for more than 600 generations. Following these crosses the sexes seemed locally adapted to each other rather than showing a random pattern. Interestingly, the fitness effect on females was not always negative after a between-strain cross, a result which was suggested to reveal the conflict load placed on females by more local males. In the present study we used populations from two regions to account for population history. A recent phylogenetic analysis of *C. heterophylla* further indicates that these two regions represent separated clades (unpubl. data, B. Baldwin et al.). Population relatedness indeed mattered when interpreting the outcome of the crosses because the ability to induce stigma receptivity in a foreign population was different depending on how closely related the foreign population was. In the analysis taking region of origin into account (Analysis 2) pollen donors were generally better at inducing stigma receptivity in crosses between populations compared to crosses within populations. This result is in line with traditional sexual conflict theory because recipients withstand donors of their own population better than donors of other populations [8], [11], [32]. On the other hand, we found that the regional origin of recipient and pollen donor significantly affected both timing of stigma receptivity and subsequent seed set. Pollen donors were less capable of inducing stigma receptivity on recipients of the other region. At the same time, reduced seed production as an indication of recipient cost was absent in crosses between regions. The reduced performance of donors in between-region crosses could be interpreted to contradict the predictions of sexual conflict theory. Alternatively, the results fit well with the idea that females should be released from the cost of interacting with local males when crossed with a foreign male, a pattern consistent with a history of sexually antagonistic coevolution within populations [40].

The fitness cost is an important parameter when inferring the relative importance of sexual conflict and other types of selection [2], [36], [38]. For the cost to be outweighed by any potential indirect benefit of superior offspring, e.g. “sexy sons” or “good genes” [54]–[55], [reviewed in 56], the indirect benefit must be significantly greater than the cost. In plants a seed size-number trade off could affect the indirect benefits; if few seeds are produced these often are larger and more nutritious [e.g. 57]. However, as the difference in seed set between early fertilisation and late fertilisation was almost three-fold in our study, it is unlikely that any indirect benefit is greater than the cost. We may, however, have overestimated the cost when cutting off the pistil if this prevented germination of more pollen at later floral developmental stages. As the cost of a lowered seed production was absent in crosses between regions, full seed set could result at early floral development even when the pistil was cut off. At this point, we do not know what caused the reduction in seed set. It is possible that only a few pollen grains were able to germinate on the partly receptive stigma, or that many ovules were not ripe enough for seeds to develop. Other possible explanations for the difference in seed set are effects of outbreeding depression, inbreeding-avoidance or heterosis [31]. Attia and Tregenza [52] for example argue that because females gained fitness in crosses between populations of the flour beetle *Tribolium castaneum*, inbreeding-avoidance might be more important than divergence caused by sexual conflict. However, as we found no significant main effect on seed set, outbreeding/inbreeding effects are less likely. We can not completely exclude effects of heterosis as seed set in fully receptive pistils may have been constrained by other factors, e.g. nutrients. On the other hand, the pronounced difference seen between regions but not between populations within the same region, does not point strongly towards heterosis.

The intraspecific phylogeny of *C. heterophylla* appears to follow the Transverse mountain ranges (unpubl. data, B. Baldwin et al.), one of the geological activity zones along which many species of the California Floristic Province have been shown to diversify and speciate [44]–[45]. These observations point towards a role for population isolation in promoting divergence, either by way of genetic drift, local ecological adaptation or other selection pressures such as sexual conflicts [7], [11], [12]. In this study, the two investigated regions of *C. heterophylla* differed in flower size and pollen tube growth rate, a pollen trait probably unrelated to the ability to induce stigma receptivity [20]. Onset of stigma receptivity showed a general trend towards being later in the south, a pattern also observed in a recent field study of 13 populations (unpubl. data, Å. Lankinen and J. Madjidian). General measures of fitness varied at a more local scale, i.e. among populations within regions. To the extent that selection has caused or contributed to these patterns, it seems that timing of stigma receptivity, flower size and pollen tube growth rate have responded to climatic or other large-scale factors [58]–[59] whereas sporophytic fitness traits reflect adaptation to local habitat conditions [60]–[62]. Because timing of stigma receptivity seems to be more similar within regions than between regions, this may partly explain why crosses between and within regions yielded different results. It is for example possible that pollen donors of the different regions have evolved different levels, or types, of sexually antagonistic traits than the pollen donors of the recipient's own population. In our case it could be hypothesised that *C. heterophylla* in the two regions are too diverged in traits related to mating system, so that pollen fail to induce stigma receptivity on recipients of other regions. Indeed, it is known that chemicals on the pollen coat can influence floral development [63] and evolve rapidly [64]. Furthermore, there is ample evidence of interactions between pollen and style mediated by intercellular communication systems [reviewed in 65], making it plausible that female sexually antagonistic traits can evolve as rapidly as male sexually antagonistic traits. So far, however, we have not identified a specific recipient sexually antagonistic trait.

*C. heterophylla* has a mixed mating system, i.e. a combination of selfing and outcrossing [66], where self-pollination occurs as a delayed selfing mechanism. More outcrossing species of the genus *Collinsia* show both later timing of selfing and delayed stigma receptivity [49], while no such correlation has been found among plants within populations, neither in this study nor previously [67]. Delayed stigma receptivity may enhance pollen competition [68] and could be a way to acquire advantages related to the mixed mating system. It has for example been shown in *C. heterophylla* that sorting among self pollen could reduce levels of inbreeding-depression in the progeny generation [69]–[70]. An interesting question worth pursuing in the future is therefore how a benefit of delayed stigma receptivity related to the mating system, would influence sexually antagonistic coevolution resulting from a sexual conflict over timing of stigma receptivity. For example, if the advantage of avoiding fertilisation by low quality self pollen would outweigh costs of induced stigma receptivity, this could prevent selection for recipient counteracting sexually antagonistic traits.

We have shown that across all our four populations of *Collinsia heterophylla* some pollen donors can fertilise ovules ahead of others, indicating the potential for sexual conflict over stigma receptivity [20]. Furthermore, onset of stigma receptivity was affected by the geographic distance between the pollen recipient and pollen donor, indicating the importance of recognising population history when inferring population interactions. Pollen donors did less well at inducing stigma receptivity on recipients from another region, at the same time as the recipient fitness cost of producing fewer seeds at early floral development disappeared. Our results are in line with the idea that recipients seem to be released from the cost of interacting with local pollen donors when crossed with a distantly related pollen donor, thus revealing antagonistic coevolution within populations. We suggest that sexual conflicts indeed may have important evolutionary consequences in plants, as well as in animals. Ultimately, studying sexual conflicts in plants may not only lead to an increased understanding of plant evolution and speciation [16], [21], but may also contribute to the whole research field of sexual selection and sexual conflict.

## Materials and Methods

### Plant material

*Collinsia heterophylla* Buist (Plantaginaceae), Chinese houses, is an annual hermaphrodite native to the California Floristic Province, North America [71]–[72]. The most common pollinators are native bees, mainly members of *Osmia*, *Bombus* and *Anthophora* [49]. The species flowers between March and June depending on elevation and latitude. Flowers are arranged in whorls on spikes and are zygomorphic with a five-lobed corolla divided into one upper and one lower lip. Corolla colour can be white to pale purple on the upper lip and dark or pale purple on the lower lip (pop. 1, 2 and 4 in this study). Some populations are polymorphic for upper-lip colour so that some plants are white and others have a dark purple band on the upper-lip (pop. 3 in this study) [73]. A flower has four epipetalous stamens and one pistil, containing up to 16–19 ovules [49, unpubl. data, J. Madjidian and Å. Lankinen]. When a flower opens the anthers are undehisced and the pistil is short. Anthers will then dehisce one at a time over 3–4 d., while the style elongates and the stigma becomes receptive. *C. heterophylla* has a mixed mating system, i.e. a combination of outcrossing and selfing [49]. Self pollination occurs at a late floral developmental stage as the style elongates and the receptive stigma comes into contact with the dehisced anthers. Estimates of mean population outcrossing rates range from 0.32 to 0.64, based on allozyme markers [74], and up to 0.94±0.27 based on morphological markers [73]. Ovaries develop into dry dehiscent seed capsules containing 2–3 mm long seeds.

Plant material of the four populations used in this study originated from Sisar Canyon (Ventura County) (pop. 1), Santa Monica Mountains (Ventura County) (pop. 2), Ferguson Ridge (Mariposa County) (pop. 3) and Hornitos Road (Mariposa County) (pop. 4). Hereafter we refer to population 1 and 2 as Southern region populations and population 3 and 4 as Northern region populations. Distances between populations in different regions (ca 350 km) and between populations within regions (pop.1–2: ca 40 km; pop. 3–4: ca 30 km) are too long for gene flow to be of any major importance.

Seeds were collected from the field by maternal family. Plants raised from these seeds were grown in a pollinator-free greenhouse and intercrossed, within populations, to obtain outcrossed plants for the crossing experiment. Experimental plants were grown in the greenhouse during the spring/early summer of 2006.

### One-donor crosses within and between populations

We performed controlled one-donor crosses on emasculated flowers within and between our four populations in order to investigate how recipient and pollen donor as well as their origin influenced how early the stigma became receptive. Flowers were emasculated to exclude self pollination. Crosses were performed at each of four successive stages of floral development, where stage 1 was represented by one dehisced anther and stage 4 by four dehisced anthers. These stages approximately correspond to day 1–4 after flower opening [following 49]; [see also 20]. Day 0 correspond to the day of flower opening, when the stigma is still unreceptive [67]. Emasculations (on day 0) were performed each day during four consecutive days, so that a full series of crosses (stage 1–4) could be conducted at the same occasion on each plant. Emasculations on newly opened flowers as well as crossings were performed at approximately the same time each day. The temperature in the greenhouse was fairly constant during the course of the experiment (4.5 weeks during April–May 2006).

Hand-pollination was carried out by adding mixed pollen from 2–3 flowers on a single plant to the stigma from a microscopic slide until the stigma was completely covered with pollen. Four hs after hand-pollination, part of the pistil was cut off (the stigma and half the style cross-section) in order to ensure that seeds were formed only if the stigma was receptive at the time of the cross [see 21 for a more detailed description of this methodology]. The time period of 4 h allows pollen tubes to reach well beyond half the style of receptive pistils [unpubl. data, Å. Lankinen, J. Maad, and W.S. Armbruster, see also 20].

**Figure 4:**
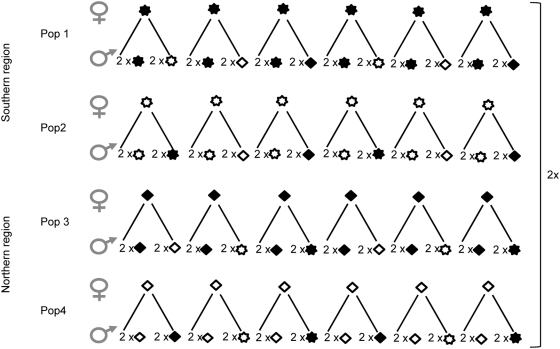
Experimental setup. One-donor crosses within and between four populations of *Collinsia heterophylla* originating from two regions. Southern region: 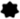 = Pop 1, 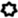 = Pop 2; Northern region: 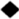 = Pop 3, 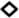 = Pop 4. ♀ represents the six recipients per population while ♂ represents the 16 donors (four of each population). Recipients of each of the four populations were crossed with donors of its own population and with donors of the three other populations. Within a population, each recipient was crossed with two donors of its own population and two donors of one foreign population. Each donor was crossed with two recipients per population. (See text for further details).

Recipients of each of our four populations were crossed with donors of its own population and with donors of the three foreign populations (Figure 4). Altogether, four pollen donors and six recipients were used per population (resulting in a total of 16 donors and 24 recipients). Within a population, each recipient was crossed with two donors of its own population and two donors of one foreign population (in alternating order across recipients). This design allowed each pollen donor to be crossed with two recipients per population (Figure 4). All crosses were replicated at least twice at the four floral developmental stages bringing the minimum total number of crosses to 768 (4 pop×6 recipients×4 donors×4 stages×2 replicates). Each recipient was hand-pollinated over a period of 2–3 weeks. Recipients were generally emasculated during the course of four consecutive days and on the fifth day we conducted crosses at stages 1–4. In most cases, we performed all crosses between a given donor by recipient combination, i.e. crosses at all four developmental stages, on the same day.

We noted when a cross resulted in a seed capsule and collected the capsules when ripe. We counted the seeds in each capsule to get an indication of recipient costs of early fertilisation.

### Measurements of sporophytic and gametophytic traits

In order to investigate how our four populations varied within and between regions and to get an indication of possible population differentiation, we measured a suite of sporophytic and gametophytic traits on plants from the four populations. As general measures of sporophytic fitness we assessed the proportion of germinated seeds, recorded the day of first flowering and counted the shoots on each individual. Further, we estimated the *in vitro* pollen performance of each pollen donor by assessing pollen tube growth rate in Hoekstra germination medium [75], based on a previously observed correlation between *in vivo* and *in vitro* pollen tube growth in this species [76]. Pollen was added to a drop of the medium on a microscopic slide. After 1h and 45 min in darkness at a constant temperature of 20°C we halted the growth by adding a drop of 100% glycerol. We measured the length of the first 10 pollen tubes observed under a light microscope and used these measurements to calculate the mean pollen tube length for each pollen donor.

In order to investigate whether mating-related traits differed between populations and regions and if timing of stigma receptivity was correlated with timing of self pollination, we measured flower size, timing of stigma receptivity, and timing of anther-stigma contact (as a measure of self-pollination). We measured the length of the flower (which corresponds keel+saccate corolla tube, see [49]) as an indication of flower size. We determined onset of stigma receptivity in a drop of 3% hydrogen peroxidase [77]. Stigmatic peroxidase activity, manifested as bubble production within 2–3 minutes, indicates stigma receptivity and has been shown to correlate with the presence of pollen tubes in pistils of *C. heterophylla* flowers [67]. We analysed stigmatic peroxidase activity twice at all five floral developmental stages (0–4) in one individual per sibling group (unit of measurement = developmental stage). We determined timing of anther-stigma contact by recording the developmental stage when anther and stigma first came into contact.

We performed most measurements on siblings of individuals used in the crossing experiment and on progeny representing a few additional maternal families: for traits measured prior to the experiment, we also included plants used in the crossing experiment. In total we used between 8 and 21 maternal families per population (3–4 plants per sibling group unless otherwise stated).

### Data analysis

We used a nested, factorial ANOVA to determine whether the timing of stigma receptivity following one-donor crosses was affected by the population from which the recipient or donor originated, the individual plant serving as recipient or donor (nested within recipient or donor population), the recipient population×donor population interaction and the recipient×donor interaction, with all factors considered as random (Analysis 1). If foreign pollen generally is better at inducing stigma receptivity than local pollen, we would expect to find a significant effect of the latter interaction. We used the same data set in another ANOVA (mixed model) to examine how timing of stigma receptivity was affected by the region (random factor) of the recipient or donor, the cross type (within/between populations) (fixed factor), the recipient region×donor region interaction and the recipient region×cross type interaction (Analysis 2). In this test, we aimed to analyse not only the general effect of relative performance of foreign vs. local pollen, but also the effect of regional differences between recipients and donors. Because neither of these two main analyses could differentiate the effect of how local vs. foreign pollen performed within a region, we made an additional two-way ANOVA (mixed model) only involving crosses within regions. We included region (random factor), the cross type (within/between populations) and their interaction in the model.

Because the results of our crosses indicated that the ability to induce stigma receptivity in a foreign population may be different when the foreign population was more distant (see results), we also examined if recipient costs of early fertilisation differed between crosses within or between regions. Using a two-way ANOVA, we investigated how recipient costs in terms of seed production was affected by the crossing stage (the developmental stage at which the flower was pollinated), cross type (within/between regions), and the interaction between crossing stage and cross type (within/between regions). In the analysis we thus pooled the data for local and foreign pollen within a region. We judge this is justified as seed set was unaffected by origin of the pollen donor within regions (two-way ANOVA: floral developmental stage *F*_3,324_ = 6.71, *P*<0.0001, cross type (own/other population within region) *F*_1,324_ = 0.37, *P* = 0.55, stage×cross type *F*_3,324_ = 0.23, *P* = 0.88).

To determine whether gametophytic and sporophytic traits differed between populations and/or regions we subjected each variable to random-effect ANOVA with region and population (nested within region) as group variables (means of maternal families or plant individuals consist of individual data points in this analysis). We also estimated population means for all variables. All variables apart from proportion of germinated seeds and timing of anther-stigma contact were approximately normally distributed. Proportion of germinated seeds was arcsine transformed to achieve normality, but we were not able to transform timing of anther-stigma contact successfully; however, as the distribution was not skewed, this should pose no serious problem [78]. Population estimates of timing of stigma receptivity and timing of anther-stigma contact were calculated as the stage at which 50% of the plants had a receptive stigma or had anther-stigma contact, respectively, using logistic regression (PROBIT procedure, SPSS 14.0). To study the relationship between timing of stigma receptivity and anther-stigma contact we performed an ANCOVA with timing of stigma receptivity as the dependent variable, population as a random factor and timing of anther-stigma contact as a covariate. We also included the interaction between population and the covariate, but as it was not significant (*P* = 0.86) we excluded it from the model.

We used type III sums of squares in all ANOVAs and performed all statistical analyses with SPSS 14.0 [79].
